# A Promising Potential of Brown Algae *Sargassum polycystum* as Irreversible Hydrocolloid Impression Material

**DOI:** 10.3390/md20010055

**Published:** 2022-01-06

**Authors:** Nurlindah Hamrun, Bahruddin Talib, Muhammad Ruslin, Hasminar Pangeran, Mochammad Hatta, Erni Marlina, Andi Sitti Hajrah Yusuf, Takashi Saito, Keng-Liang Ou

**Affiliations:** 1 Department of Oral Biology, Faculty of Dentistry, Hasanuddin University, Makassar 90245, Indonesia; 2 Department of Prosthodontic, Faculty of Dentistry, Hasanuddin University, Makassar 90245, Indonesia; bahruddintalib@unhas.ac.id; 3 Department of Oral and Maxillofacial Surgery, Faculty of Dentistry, Hasanuddin University, Makassar 90245, Indonesia; mruslin@unhas.ac.id (M.R.); hajrahyusuf@unhas.ac.id (A.S.H.Y.); 4 Dental Hospital, Hasanuddin University, Makassar 90153, Indonesia; hasminarpangeran@gmail.com; 5 Molecular Biology and Immunology Laboratory, Faculty of Medicine, Hasanuddin University, Makassar 90245, Indonesia; hattaram@yahoo.com; 6 Department of Oral Medicine, Faculty of Dentistry, Hasanuddin University, Makassar 90245, Indonesia; erni.marlina@unhas.ac.id; 7 Division of Clinical Cariology and Endodontology, Department of Oral Rehabilitation, School of Dentistry, Health Sciences University of Hokkaido, Hokkaido 061-0293, Japan; t-saito@hoku-iryo-u.ac.jp (T.S.); klou@tmu.edu.tw (K.-L.O.); 8 Biomedical Technology R&D Center, China Medical University, Taichung 404, Taiwan; 9 Department of Oral Hygiene Care, Ching Kuo Institute of Management and Health, Keelung 203, Taiwan; 10 3D Global Biotech Inc. (Spin-off Company from Taipei Medical University), New Taipei City 221, Taiwan; 11 Taiwan Society of Blood Biomaterials, New Taipei City 221, Taiwan; 12 Department of Dentistry, Shuang Ho Hospital, Taipei Medical University, New Taipei City 235, Taiwan

**Keywords:** accuracy, *Sargassum polycystum*, impression material, sodium alginate, irreversible hydrocolloid

## Abstract

This study aimed to investigate the potential use of brown algae *Sargassum polycystum* as irreversible hydrocolloid (alginate) impression material. Potassium alginate extracted from *Sargassum polycystum* was prepared in three different compositions (14%, 15%, and 16%) and mixed with other standard components to form an alginate impression material. Prior to that, the purity of potassium alginate was quantified with Fourier Transform Infrared Spectroscopy (FTIR) analysis. As a control material, the alginate impression material from a commercially available product was used. All alginate impression materials were then applied to a die stone model. Dimensional accuracy was measured by calculating the mesiodistal width of incisors in the generated dental cast using a digital caliper 0.01 accuracy (five replications). In addition, to evaluate the dimensional stability, the impression results were poured at four different periods (immediately, 5 min, 10 min, and 15 min). An independent *t*-test was performed to compare the measurement results with *p* < 0.05 considered significant. Analytical results confirm that the impression material containing 15% potassium alginate gives the best dimensional accuracy similar to control (*p* > 0.05). Meanwhile, the optimal dimensional stability was produced in the impression material containing 16% potassium alginate. Our study suggested that brown algae *Sargassum polycystum* has a promising potential to be used as an alginate impression material in clinical application.

## 1. Introduction

Impression procedure is one of the most crucial steps in producing successful dental appliances, indirect restorations, and prostheses particularly in the orthodontic and prosthodontic field [1,2,3]. The impression material is especially beneficial for molding serrated jaws or partial edentulous jaws to obtain a dental cast for master study [1,4]. A precise master study is essential to complete treatment planning and the preliminary phase of dental appliance [1,5,6]. It is generally known that the marginal fit of final prosthesis or indirect restoration depends largely on how meticulously the impression material visualizes the dentition and surrounding oral mucous [3,5]. Any flaws in this stage may have consequences in a poorly fitting prosthesis [3,7]. Thus, it is mandatory for the impression material to accurately reproduce the detail of intraoral hard and soft tissues to form a precise dental cast.

Ideal criteria for impression material should meet these requirements: adaptive to the oral tissues, viscous enough to be loaded in the tray, set into rubbery or rigid solid in a reasonable amount of time, dimensionally stable, and biocompatible [2,8]. Recently, irreversible hydrocolloid (alginate) is the most popularly used impression material in dental field owing to its excellent clinical and physical properties including ease of handling in processing, highly acceptable recording of details, minimum equipment necessary, and flexibility of setting time [6,9]. Moreover, alginate is considered inexpensive and well tolerated by patients, with favorable aroma and taste [1,8]. Despite the advantages of this material, the hydrophilic nature of alginate causes its dimensional stability to diminish over time due to water absorption (imbibition) and water release (syneresis) phenomena [1,2,9]. In order to prevent deformation, some studies recommended pouring the mold immediately or within a few minutes of being removed from the mouth [1,2,5,10,11]. In an attempt to argue the criteria for an ideal impression material, some researchers were keen to formulate a new irreversible hydrocolloid impression material [12,13].

The main active ingredients of irreversible hydrocolloid impression materials are sodium alginate, potassium alginate, or triethanolamine alginate [5,14]. Both sodium alginate and potassium alginate can be extracted from species of multicellular brown algae, including *Sargassum* sp., *Turbinaria* sp., *Hormophysa* sp., and *Padina* sp. [15]. The brown macroalgae are characterized architecturally by an alginophyte cell wall structure composed of ß-D-mannuronic acid and α-L-guluronic acid [16]. Alginate extracted from these species can be homogenized in water to form soluble alginate, which can react with calcium sulfate to generate insoluble alginate [6,14]. Most of the species of brown algae are prevalent in a number of maritime countries, including Indonesia [17]. Unfortunately, even though Indonesia is surrounded by a *Sargassum*-rich coastline and considered to have the potential to fulfill all the demand for an alginate-based irreversible hydrocolloids impression material, these resources are still under-cultivated [15]. Against this background, in the present study we aimed to investigate the implementation of alginates from brown algae *Sargassum polycystum* as dental impression material and compare it with commercially available alginate product. We specifically targeted the evaluation of the dimensional accuracy and dimensional stability of this material to generate optimal impression results.

## 2. Results

**Figure 1:**
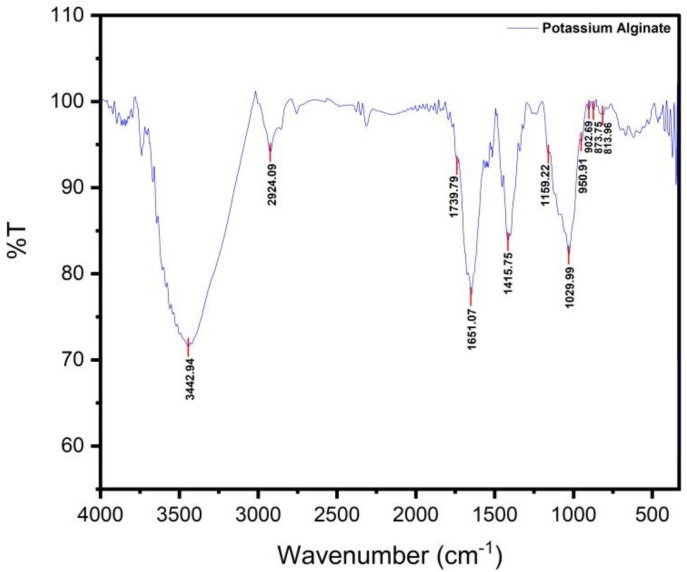
Infrared spectrum of potassium alginate extracted from *Sargassum polycystum*.

In this study, we formulated an irreversible hydrocolloid impression material composed of potassium alginate extracted from brown algae *Sargassum polycystum*. The extracted potassium alginate was blackish brown in color. Off a total 2.5 kg of dried algae, the yield potassium alginate was 12% or 300 g. As shown in Figure 1, functional group analysis revealed that the extracted potassium alginate had the same structure as the standard potassium alginate in terms of hydroxyl groups, carboxyl groups, carbonyl groups, COO bonds, C-O-H bonds, C-O-C bonds, and K-bonds.

Fourier Transform Infrared Spectroscopy (FTIR) analysis was performed to quantify the purity of potassium alginate (Table 1). Analytical results demonstrated three specific peaks associated with authentic potassium alginate, namely hydroxyl (OH) groups with an absorption area at 3700–3100 cm^−1^, asymmetrical -COO- groups with an absorption area at 1600–1590 cm^−1^, and symmetrical -COO- groups with an absorption peak at 1400 cm^−1^.

The uptake area of 3442.94 cm^−1^ illustrated the absorption of hydroxyl groups (O-H) similar to those of commercial alginates used as a standard. Uptake in areas 2924.09 cm^−1^ and 1739.79 cm^−1^ showed absorption of aliphatic C-H groups and carbonyl groups (C=O). The -COO- asymmetric and -COO- symmetrical groups were shown in the uptake area of 1651.07 cm^−1^ and 1415.75 cm^−1^. When protons are replaced by monovalent ions (potassium), the peaks appear at around 1600 cm^−1^ and 1400 cm^−1^, respectively, and were characterized by asymmetrical and symmetrical uptake of carboxyl groups free of potassium alginate. Uptake in the 1029.99 cm^−1^ and 1159.22 cm^−1^ regions showed uptake of C-OH and C-O-C groups. The guluronic fingerprint area in the sample was detected in the absorption area of 813.96 cm^−1^; 873.75 cm^−1^; 902.69 cm^−1^ while C-O uronic acid absorption was shown in the absorption area of 950.91 cm^−1^.

The spectra of the typical guluronic and mannuronic regions are markers that provide evidence of the presence of potassium alginate. The presence of hydroxyl, carboxylate, and ether, which are the main functional groups in potassium alginate, indicates that the presented characteristic spectrum resembles the standard potassium alginate absorption spectrum in several references.

Table 2 highlights the mean and standard deviation of the mesiodistal width value of incisors measured in dental cast from brown algae *Sargassum polycystum* and control material at four different time points. Results indicated that no significant differences were observed in mesiodistal width of incisor in dental cast made from potassium alginate with concentrations 14%, 15%, and 16% compared to control at all casting times (*p* > 0.05). Based on the mesiodistal width of incisors, it can be seen that the closest measurement value to the master model (8.000 mm) was obtained in the dental cast imprinted with 15% potassium alginate (7.993 mm) and control (7.996 mm). These findings implied that alginate material made from *Sargassum polycystum* with 15% concentration can reproduce a casting model as accurate as the readily available product.

**Figure 2:**
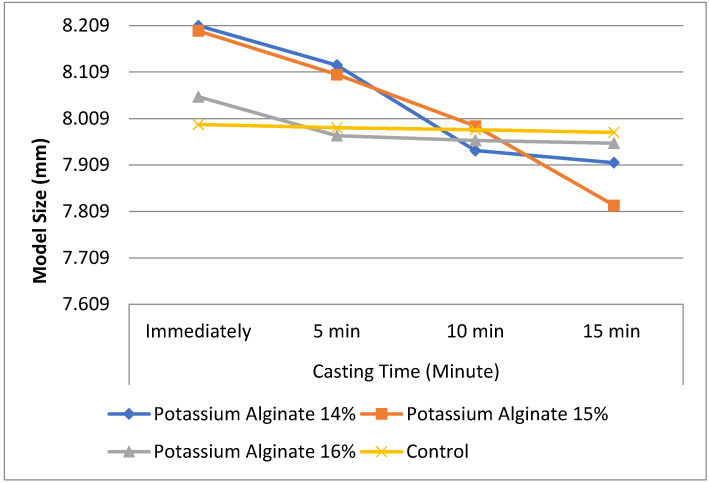
Comparison of accuracy measurement of different potassium alginate concentration formulas (14%, 15%, 16%, and control) at different casting times.

Figure 2 depicts the accuracy measurement of potassium alginate in concentrations of 14%, 15%, 16%, and control obtained after several casting times. It was observed that the control alginate has consistent and steady values from the start of measurement time (immediately) until the end of measurement time (15 min). The measurement results of the impression material from *Sargassum polycystum* with 16% concentration appeared to be closest to the control. This finding reported that alginate impression material containing 16% potassium alginate from *Sargassum polycystum* provides the optimal dimensional stability, similar to that of the commercially available product.

## 3. Discussion

Potassium alginate is a common component contained in standard dental impression materials. In this study, the alginate impression material was composed of potassium alginate extracted from brown algae *Sargassum polycystum*. The sample spectrum was identified with FTIR analysis on a transmission basis for quantitative estimation. Based on the functional group analysis, we found that the extracted potassium alginate had the same structure as the potassium alginate demonstrated in several references [18,19]. According to Ju et al. [18], alginate compounds have three specific absorption peaks corresponding to hydroxyl (OH) groups at around 3700–3100 cm^−1^, -COO-asymmetrical groups at around 1600–1590 cm^−1^, and -COO-symmetrical groups at around 1400 cm^−1^ [18]. This standard was in accordance with a previously published study indicating the typical absorption of potassium alginate patterns of hydroxyl (OH), asymmetrical and symmetrical -COO- groups, and guluronic and mannuronic acid, and was the basis for concluding that the material isolated was potassium alginate [19].

In the present study, we quantified the dimensional accuracy of the impression material based on the measurement value of dental cast. We found that the impression material containing 15% potassium alginate showed a dimensional accuracy comparable to that of the control material. Our results are in agreement with those of McCabe et al. [20] who suggested that potassium alginate with a concentration of 11–16% was a suitable components for an irreversible hydrocolloid [20]. Moreover, other studies reported that the 15% concentration of potassium alginate was appropriate to formulate an irreversible hydrocolloid impression material [21,22].

The capacity of sodium alginate to create viscous solutions and gels in aqueous conditions is important in the pharmaceutical industry [17]. This concept has also been applied in dental impression formulations since a low viscosity (low polymerization degree) of alginate will not form a gel [16,22]. Potassium salt and calcium sulfate serve as the reactive constituents in the alginate impression material, allowing the material to form a gel when mixed with water. In most cases, some commercially available products were added with filler and other proprietary ingredients to maintain setting time, consistency, strength, elasticity, and dimensional stability [1]. As a rule of thumb, the concentration value will be greatly influenced by the molecular weight of the alginate. The higher the molecular weight, the higher the viscosity [2,14]. Previously published studies reported that the viscosity of dental impression material can be increased if the extraction of potassium alginate is carried out using freshly harvested seaweed [16,23,24]. However, it is generally believed that the exact proportions of the dental impression formula vary according to the type of alginate used [22,25,26].

In our experiment, the dimensional stability was measured by observing the accuracy of the reproduced model at different time points (0, 5, 10, and 15 min) after the impression was made. Results presented in the current study indicated that alginate impression material containing 16% potassium alginate from *Sargassum polycystum* provides the optimal dimensional stability, similar to that of the commercially available product, although it still showed a slight tendency to be smaller than the original tooth. The dimensional stability of the stone model is greatly affected by the casting time, specifically because of the expansion caused by imbibition and shrinkage due to water evaporation from the irreversible hydrocolloid impression materials [1,2]. Previously published studies agreed in hypothesizing that the maximum accuracy could be achieved by casting the impression 10 min after elastic recovery [26,27]. Their results were supported by Nassar, [20] who found that casting for less than one hour did not cause any significant alterations in dimensional terms. However, he reported a significant difference of 1% for casting performed for 4 h or longer [1,10,11,27].

Besides casting time, other factors that contribute to the accuracy and dimensional stability of irreversible hydrocolloid material are the room temperature, the water temperature of the mixture impression, the water-to-powder ratio, and the stirring intensity [1,6,11,14]. Theoretically, a warmer water temperature or general room temperature might hasten the setting time, whereas colder water will slow down the setting time. The ideal temperature to use in the mixing process is 73 °F (22 °C) or as close to room temperature as possible [28]. In addition, different powder and liquid compositions will also affect alginate properties. A thin dough will shorten the setting time, while a thicker one will reduce the setting time due to less flexibility [14]. Thus, the water added in the mixing process must be proportional to the powder used so that the ideal gel consistency can be obtained in a short setting time [1,29].

Another factor that contributes to the accuracy and stability of impression material is the stirring intensity. Higher stirring intensity and rate will hasten the setting time and vice versa. However, prolonged stirring may break up the woven calcium alginate gel and reduce its strength [30]. Manual stirring of alginate material is usually carried out using a figure-eight movement at a fast speed while pressing the mixture against the walls of a rubber bowl. The mixing should be performed with an intermittent (180°) inversion of the spatula to prevent the entrapment of air bubbles. Those technical instructions may improve the quality of the alginate impression material in the mortar [27,31,32].

Considering some factors that can affect dimensional accuracy and stability of impression materials, the present study is limited in controlling several issues, such as the low resistance of the generated formulation in terms of temperature. This led to a need for more conditioning of the impression material after storage and caused less solidification, as well as fragile impressions. Nevertheless, results in the current study strongly demonstrate the dimensional stability of impression material made from *Sargassum polycystum*, specifically that formulated in 16% alginate concentration.

## 4. Materials and Methods

### 4.1. Extraction of Potassium Alginate from Sargassum polycystum

The sample used in the present study was brown algae species, *Sargassum polycystum* taken from Punaga and Puntondo coast, Takalar, South Sulawesi, Indonesia. As much as 2.5 kg of dried *Sargassum polycystum* was taken to extract potassium alginate. The extraction procedure of potassium alginate was started with the immersion process of dried *Sargassum polycystum* in 1% hydrochloric acid (HCl) for 1 h with a ratio 1:1.5 water/volume (*w*/*v*). After that, *Sargassum polycystum* was washed with clean water to restore the neutral pH.

The alginate was extracted by homogenizing the *Sargassum polycystum* in 2% potassium carbonate (K_2_CO_3_) with ratio 1:1.5 (*w*/*v*), and subsequently heated at 60–70 °C for 2 h. After that, the material was let to cool down. The homogenized material was then blended and heated again in 2% K_2_CO_3_ and finally the cooling step was repeated. The material was filtered and subjected to a bleaching step by immersion in 4% sodium hypochlorite (NaOCl) for 1 h. After that, the alginate was precipitated by treatment with 10% HCl to achieve a pH = 2.8–3.

The alginate deposits were converted to potassium alginate by adding 10% K_2_CO_3_ to reach a neutral pH (pH = 7). Separation was then completed by gradually and continuously mixing and stirring the material and then leaving it at room temperature for 30 min. After that, the alginate material was stored in petri dishes to dry for 2 days. Lastly, the alginate material was landed and filtered through a 200 mesh sieve to obtain a potassium alginate powder [1,7,8].

### 4.2. Purity Test by FTIR Analysis

In order to identify the extracted material as potassium alginate, a purity test was performed by FTIR (Shimadzu FTIR-8400S) analysis. Analytical results from the extracted potassium alginate were compared to a standard value. The potassium alginate powder was pulverized using a mortar and pestle, filtered, and combined with potassium bromide (KBr) powder to create KBr discs. The discs were created using a mini hand press connected to a 720 mmHg pressure tension vacuum pump for 5 min. Finally, the FTIR spectra were analyzed by a computer connected to the FTIR instrument [9,10].

### 4.3. Producing Irreversible Hydrocolloid Impression Material

The impression material was generated by mixing the ingredients using a mortar and pestle. The impression material was prepared in three different concentrations of potassium alginate (14%, 15%, and 16%). Other ingredients that were mixed are 16% calcium sulfate, 3% potassium titanium fluoride, 2% trisodium phosphate, 60% diatomaceous earth, and 4% zinc oxide. This formulation was according to the standard ingredients for the irreversible hydrocolloid impression material as introduced in previously published studies [1,2,11].

In this study, the irreversible hydrocolloid impression materials made from *Sargassum polycystum* (14%, 15%, and 16%) were compared with commercially available product (Aroma fine plus normal set, GC, Japan) which is generally used in dental practice in our region. This impression material consists of 15% potassium alginate, 16% calcium sulfate, 4% zinc oxide, 3% potassium fluoride, 60% diatomaceous earth, and 2% sodium sulfate.

### 4.4. Impression Procedure

The master model used in this study was a die stone model with one maxillary right first incisor. The mesiodistal width of the master model is 8.0 mm. The tooth was embedded in the cast at the base to obtain an evenly distributed pressure during the molding process. A cutting die was prepared as described in American Standard Test and Material document D-1004-94a. For the impression procedure, the impression material powder was homogenized with water with the composition of 8 g powder to 12 mL water for 60–90 s. The mixing process was done on a vibrator with 2–3 min of working time was set. The master model was imprinted, and 5–6 min of setting time was imposed. This process will allow the impression to set before being removed from the mold.

### 4.5. Casting Procedure

The generated mold from impression process was casted at four different time periods including immediately, 5 min, 10 min, and 15 min under strictly controlled laboratory conditions. Dental stone powder type IV was homogenized on a vibrator at ratio of 50 g to 12.5 mL water for 60 min to produce a dental cast. The dental cast was removed from the tray at least 30 min after casting process for allowing the dental stone to dry [2,8,13]. In total, 80 samples were made (five samples for each alginate materials from each period).

### 4.6. Model Measurement

For the evaluation of dimensional accuracy of the impression materials, a measurement procedure was conducted in the dental cast using a digital caliper (Krisbow, Indonesia, precision rate 0.01 mm). The reference point was set at two areas: the distal tips of maxillary right first incisor and the mesial tips of maxillary right first incisor. The measurements of the master model and the dental stone were carried out in five replications. All impressions, casts, and measurements were made by the same operator to avoid bias. The measurement values were presented as mean and standard deviation.

### 4.7. Statistical Analysis

The data were analyzed using SPSS version 17 with an independent sample *t*-test. A value of *p* < 0.05 was considered statistically significant. We tested differences between the estimated means and the true values at α = 5%.

## 5. Conclusions

Within the limitation of this study, we can conscientiously conclude that potassium alginate derived from brown alga *Sargassum polycystum* can be used as an alternative material for irreversible hydrocolloid impression material in daily dental practice. Potassium alginate with 15% concentration provides the most accurate impression, while potassium alginate with 16% concentration presents the most stable impression up to 15 min. For future study, more research is required to characterize the behavior and nature of this material.

## Figures and Tables

**Table 1:** Functional group analysis in potassium alginate extracted from *Sargassum polycystum*.

Peak Wavelengths for Potassium Alginate Extracted from *Sargassum polycystum* (cm^−1^)	Functional Group Interpretation	Wavelength Reference (cm^−1^)
3442.94	Hydroxyl (-OH)	3700–3100
2924.09	C-H aliphatic	3000–2800
1739.79	Carbonyl (C=O)	1870–1650
1651.07	COO-asymmetric	1600–1590
1415.75	COO-symmetrical	1410
1029.99	C-OH	1300–1000
1159.22	C-O-C	1250–1170
813.96; 873.75; 902.69	Guluronic fingerprints	890–900
950.91	C-O stretching Uronic acid	948

**Table 2:** The measurement value of mesiodistal width of incisors obtained from impression material contained 14%, 15% and 16% of potassium alginate, and control based on the casting time.

Casting Time (Minutes)	Mesiodistal Width			Mesiodistal Width			Mesiodistal Width			*p*-Value
	PA 14% (mm)	Control (mm)	Mean Difference	PA 15% (mm)	Control (mm)	Mean Difference	PA 16% (mm)	Control (mm)	Mean Difference	
	Mean ± SD	Mean ± SD		Mean ± SD	Mean ± SD		Mean ± SD	Mean ± SD		
Immediately	8.209 ± 0.267	7.996 ± 0.005	0.213	8.197 ± 0.001	7.996 ± 0.005	0.201	8.056 ± 0.561	7.996 ± 0.005	0.060	0.148
5	8.124 ± 0.133	7.988 ± 0.010	0.135	8.104 ± 0.119	7.988 ± 0.010	0.115	7.971 ± 0.053	7.988 ± 0.010	−0.017	0.084
10	7.940 ± 0.254	7.985 ± 0.005	−0.045	7.993 ± 0.441	7.985 ± 0.005	0.008	7.961 ± 0.149	7.985 ± 0.005	−0.023	0.715
15	7.914 ± 0.147	7.978 ± 0.009	−0.065	7.822 ± 0.183	7.978 ± 0.009	−0.156	7.956 ± 0.067	7.978 ± 0.009	−0.022	0.381

PA = Potassium Alginate, Independent *t*-test (*p* < 0.05).

## Data Availability

The data presented in this study are available on request from the corresponding author.
